# A comparison of techniques for classifying behavior from accelerometers for two species of seabird

**DOI:** 10.1002/ece3.4740

**Published:** 2019-02-21

**Authors:** Allison Patterson, Hugh Grant Gilchrist, Lorraine Chivers, Scott Hatch, Kyle Elliott

**Affiliations:** ^1^ Department of Natural Resource Sciences McGill University Ste Anne‐de‐Bellevue Quebec Canada; ^2^ Environment and Climate Change Canada National Wildlife Research Centre Ottawa Ontario Canada; ^3^ Institute for Seabird Research and Conservation Anchorage Alaska

**Keywords:** accelerometer, animal behavior, behavioral classification, movement ecology, *Rissa tridactyla*, seabird tracking, *Uria lomvia*

## Abstract

The behavior of many wild animals remains a mystery, as it is difficult to quantify behavior of species that cannot be easily followed throughout their daily or seasonal movements. Accelerometers can solve some of these mysteries, as they collect activity data at a high temporal resolution (<1 s), can be relatively small (<1 g) so they minimally disrupt behavior, and are increasingly capable of recording data for long periods. Nonetheless, there is a need for increased validation of methods to classify animal behavior from accelerometers to promote widespread adoption of this technology in ecology. We assessed the accuracy of six different behavioral assignment methods for two species of seabird, thick‐billed murres (*Uria lomvia*) and black‐legged kittiwakes (*Rissa tridactyla*). We identified three behaviors using tri‐axial accelerometers: standing, swimming, and flying, after classifying diving using a pressure sensor for murres. We evaluated six classification methods relative to independent classifications from concurrent GPS tracking data. We used four variables for classification: depth, wing beat frequency, pitch, and dynamic acceleration. Average accuracy for all methods was >98% for murres, and 89% and 93% for kittiwakes during incubation and chick rearing, respectively. Variable selection showed that classification accuracy did not improve with more than two (kittiwakes) or three (murres) variables. We conclude that simple methods of behavioral classification can be as accurate for classifying basic behaviors as more complex approaches, and that identifying suitable accelerometer metrics is more important than using a particular classification method when the objective is to develop a daily activity or energy budget. Highly accurate daily activity budgets can be generated from accelerometer data using multiple methods and a small number of accelerometer metrics; therefore, identifying a suitable behavioral classification method should not be a barrier to using accelerometers in studies of seabird behavior and ecology.

## 1 INTRODUCTION

Developments in biologging technology have greatly advanced our ability to study wildlife throughout their ranges, without restrictions and bias imposed by human observation and accessibility (Cagnacci, Boitani, Powell, & Boyce, 2010; Hebblewhite & Haydon, 2010). Traditional methods for measuring animal activity involve direct observation of animals in the field, which is labor intensive. Direct observation limits the scale of observations to times and locations where focal species are accessible to biologists, and creates opportunity for bias if focal animals, or their predators and prey, change behavior in response to the presence of observers (MacArthur, Geist, & Johnston, 1982; Quiros, 2007). Measuring animal activity with accelerometers overcomes most of these challenges by continuously logging activity wherever the individual goes, and, if small enough, with very little impact on the animal's behavior (Wilmers et al., 2015). Accelerometers have been used to answer a wide‐range of ecological questions relating to prey capture (Sato et al., 2015), energetics (Elliott, Chivers, et al., 2014; Robson, Chauvaud, Wilson, & Halsey, 2012), physiology (Watanuki, Niizuma, Geir, Sato, & Naito, 2003), migration strategies (Bishop et al., 2015; Wiemerskirch, Bishop, Jeanniard‐du‐Dot, Prudor, & Sachs, 2016); but perhaps the most widespread application of accelerometers is in obtaining time‐activity budgets (Berlincourt, Angel, & Arnould, 2015; Brown, Kays, Wikelski, Wilson, & Klimley, 2013).

Combined with other sensors, accelerometers provide a powerful tool to understand the relationships between animal behavior, energetics, and the environment. Many GPS tracking studies infer animal behavior from path geometry, collecting locations at very high intervals to obtain detailed tracks to support inferences about animal behavior based on path trajectories (Grémillet et al., 2004; Mendez et al., 2017; Ryan, Petersen, Peters, & Grémillet, 2004; Wakefield, Phillips, & Matthiopoulos, 2009; Weimerskirch, Le Corre, & Bost, 2008). Pairing GPS and accelerometer sensors could reduce the frequency of required GPS fixes, extending the battery life for longer deployments without sacrificing detailed behavioral data. Satellite and light‐based tracking methods record locations with low temporal resolution (geolocators) and at irregular intervals (satellite transmitters), which precludes inference about detailed behavior. If these methods were coupled with accelerometers, then it would be possible to track species over large spatial scales for extended time‐periods with high temporal resolution. This type of detailed, long‐term tracking of animal movements and behaviors will allow robust inference about animal ecology and how species interact with their environments (Cagnacci et al., 2010; Wakefield et al., 2009).

The ease with which biologists can deploy tracking devices to study the movements of wild animals has exceeded the ability of biologists to categorize, analyze, and interpret the volume of data these efforts have generated. Widespread adoption of accelerometers to measure animal behavior is inhibited by limited validation, which has contributed to a lack of consensus on analysis methods. A host of methods have been proposed for classifying animal behavior from accelerometer data (Appendix S1), including movement thresholds (Brown et al., 2013; Moreau, Siebert, Buerkert, & Schlecht, 2009; Shamoun‐Baranes et al., 2012), histogram analysis (Collins et al., 2015), *k*‐means (KM) cluster analysis (Angel, Berlincourt, & Arnould, 2016; Sakamoto et al., 2009), *k*‐nearest neighbor analysis (Bidder et al., 2014), classification and regression trees (Shamoun‐Baranes et al., 2012), neural networks (NN; Nathan et al., 2012; Resheff, Rotics, Harel, Spiegel, & Nathan, 2014), random forests (Bom, Bouten, Piersma, Oosterbeek, & van Gils, 2014; Nathan et al., 2012; Pagano et al., 2017), hidden Markov models (HMM; Leos‐Barajas et al., 2016), expectation maximization (EM; Chimienti et al., 2016), and super machine learning (Ladds et al., 2017). At least three custom software applications are available for classifying animal behavior from trained accelerometer data: AcceleRater (Resheff et al., 2014), G‐sphere (Wilson et al., 2016), and Ethographer (Sakamoto et al., 2009). Many of these methods use machine‐learning techniques that are difficult to interpret because underlying processes are opaque. Numerous accelerometer‐derived metrics have been employed as predictors in classification models, often without any critical evaluation of their value in improving classification accuracy. We reviewed 15 similar studies that classified animal behavior from accelerometers, to identify common accelerometer metrics used in classifications (Appendix S1). These studies used between 1 and 147 different variables in their classification models; the median number of parameters included was seven. Using large numbers of predictor variables may make classifications unnecessarily complex, potentially discouraging biologists from adopting this tool, and make methods developed on one data set less generalizable to other studies. Simpler approaches may appear inadequate in comparison to sophisticated analyses, while many complex methods can be difficult for most ecologists to implement.

Identifying an appropriate classification technique is further complicated because most methods are based on small sample sizes, with limited or no validation of classification accuracy. In a sample of 15 studies, only 10 attempted to validate their classifications, only six had sample sizes of more than 10 individuals from the same species, and five studies used data from <5 individuals from some species for analysis (Appendix S1). Many classification methods rely on training data acquired through direct observation of free‐living (Nathan et al., 2012), domesticated (Moreau et al., 2009), or captive (Pagano et al., 2017) animals. Training data can be challenging or impossible to collect for wide‐ranging species like seabirds, with some species travelling hundreds of kilometers in a single foraging trip. Observations of captive animals are unlikely to represent the full range of animal behavior for species that move over large spatial scales (Pagano et al., 2017). There is a need for robust unsupervised classification methods and for alternative approaches to developing training and validation data sets for species, such as most seabirds, that cannot be observed directly in the wild.

We compared six different methods for classifying behavior using accelerometer data from two seabird species: thick‐billed murres (*Uria lomvia*) and black‐legged kittiwakes (*Rissa tridactyla*). In this study, we focus on comparing methods for classifying the main behaviors (flying, swimming, on colony, and diving) that comprise a daily activity budget for two seabird species. Daily activity budgets have been widely used in studies of seabird behavior (Ropert‐Coudert et al., 2004), energetics (Birt‐Friesen, Montevecchi, Cairns, & Macko, 1989), and ecology (Furness & Camphuysen, 1997); identifying robust methods for calculating daily activity budgets from accelerometer data should contribute to wider application of this technology. Accelerometer deployments were paired with GPS data loggers and GPS tracks were used to validate the accuracy of accelerometer‐based classifications. High‐resolution GPS data are already widely used for behavioral classification in free‐living birds, thus, these data provide a good option for validating classifications on a large number of individuals engaging in a full range of natural activities. Our analysis focused on identifying coarse‐scale behaviors: resting on colony, flying, swimming, and diving (for murres). Quantifying these behaviors is useful for many seabird studies and these behaviors can be inferred from high‐resolution GPS tracks. We compared overall accuracy and behavior‐specific accuracy for each species. We also considered the effect of breeding stage (incubation vs. chick rearing) on classification accuracy; although behavior in general should not change between breeding stages, the frequency of different behaviors can change, and factors such as level of activity and posture while at the nest could change, affecting our ability to accurately identify these behaviors. To determine if classification method affects estimates of energy expenditure we also used daily activity budgets from each classification to calculate daily energy expenditure (DEE). Finally, we used variable selection to assess whether or not models using more predictor variables perform better than models with fewer variables and to identify the variables that make the greatest contribution to improvements in classification accuracy for each species.

## 2 METHODS

### 2.1 Tagging methods

We deployed GPS‐accelerometers (Axy‐trek; Technosmart, Rome, Italy; 18 g) on 21 incubating and 19 chick‐rearing murres breeding at Coats Island, in 2018. Murres were captured using a noose pole and biologgers were attached to the back feathers using TESA tape (TESA 4651, Hamburg, Germany). Murres were released at the capture site and re‐captured between 2 and 4 days later to retrieve data loggers. The biologgers were programed to collect GPS locations at 1 min intervals, depth at 0.1 m resolution and 1 Hz intervals, acceleration in three axes at 25 Hz, and temperature at 1 Hz. Note that deployment of similar tags altered dive duration, flight costs, and chick feeding rates (Elliott, Davoren, & Gaston, 2007; Elliott, Vaillant, et al., 2014). As all individuals should be similarly impacted, these tag effects should not affect the results of this study.

We deployed tri‐axial accelerometers (Axy‐3; Technosmart; 3.2 g), paired with GPS biologgers (CatTraQ; Catnip Technologies, USA; 14 g), on black‐legged kittiwakes at Middleton Island, Alaska, USA, in 2013. Data were collected from 17 incubating and 19 chick‐rearing kittiwakes. Both biologgers were attached to the back feathers of kittiwakes using Tesa tape (TESA 4651). Kittiwakes were released at the capture site and re‐captured between 1 and 3 days later to retrieve data loggers. The biologgers were programed to collect GPS locations at 30 s intervals and tri‐axial acceleration at 25 Hz. Deployment of these tags had no impact on reproductive success and survival, but altered flight duration (Chivers, Hatch, & Elliott, 2016). As all individuals should be similarly impacted, these tag effects should not affect the results of this study.

### 2.2 Accelerometer‐derived metrics

We focused on three types of accelerometer‐derived metrics for behavior classifications: wing beat frequency (WBF), pitch, and dynamic acceleration. We chose variables that we thought would be related to the target behaviors based on our prior knowledge of the study species. We calculated WBF by extracting the dominant frequency in the *Z*‐axis using a Fast Fourier Transform (FFT) over a 5‐s moving window. The FFT was performed using the “fft” function in base R. The peak frequency in the *Z*‐axis can detect signals that represent motion other than flying (such as walking or sea surface waves), however, for simplicity we refer to this as WBF going forward, as this was the signal we were interested in extracting from the accelerometer. Birds in flapping flight should display characteristic frequencies in vertical motion while travelling.

Pitch measures vertical body angle based on the static acceleration (acceleration averaged over time) of all three axis (Table 1). We expected pitch to change between different behaviors, because the body angle of a bird will change between time on land, swimming, and flight. All pitch values were corrected for differences in device orientation by standardizing acceleration measurements to a pitch of 0° for periods of presumed flight (WBF between 6–9 Hz for murres and 3–6 Hz for kittiwakes) (Elliott, Chivers, et al., 2014), when all birds should have a similar and consistent body orientation (Chimienti et al., 2016; Watanuki et al., 2003).

**Table 1:** Accelerometer‐derived metrics calculated prior to behavioral classifications. Only pitch, *SD_Z_*, *SD*_ODBA_, WBF, and depth were used in classifications, other statistics shown were calculated to obtain final classification parameters

Statistic	Label	Equation	Description
Static acceleration	*S_X_*, *S_Y_*, *S_Z_*	$\frac{\sum X}{n},\frac{\sum Y}{n},\frac{\sum Z}{n}$	Average acceleration in each axis, calculated over a 2‐s moving window
Pitch	Pitch	$\tan^{-1}\left(\frac{S_{X}}{\sqrt{S_{Y}^{2}+S_{Z}^{2}}}\right)*\frac{180}{\pi }$	Vertical orientation of the body angle
Dynamic acceleration	*D_X_*, *D_Y_*, *D_Z_*,	*S_X_* *− X, S_Y_* *− Y, S_Z_* *− Z*	Residual acceleration in each axis, calculated over a 2‐s moving window
Overall dynamic body acceleration	ODBA	*|D_X_| + |D_Y_| + |D_X_|*	Dynamic acceleration summed across all three axes
Standard deviation of dynamic acceleration in *Z*‐axis	*SD_Z_*	$\sqrt{\frac{1}{N}\sum_{i=1}^{N}{\left(Dz_{i}-\frac{\sum \mathrm{Dz}}{n}\right)}^{2}}$	Variation in the dynamic acceleration in the *Z*‐axis
Standard deviation of overall dynamic body acceleration	*SD*_ODBA_	$\sqrt{\frac{1}{N}\sum_{i=1}^{N}{\left({\text{ODBA}}_{i}-\frac{\sum \text{ODBA}}{n}\right)}^{2}}$	Variation in the dynamic acceleration in the ODBA
Wing beat frequency	WBF		Dominant frequency in the *Z*‐axis, calculated using a 5‐s moving window
Depth	Depth		Meters below sea level

Dynamic body acceleration integrates the amount of dynamic acceleration (i.e., after removing the static component due to gravity and associated with posture) over a fixed time period, and can be used as an index of movement (Shepard, Wilson, Quintana, et al., 2008). Dynamic body acceleration can be measured along each axis individually, or as a composite of all three axes using overall dynamic body acceleration (ODBA, Table 1). For murres, we used standard deviation of the overall dynamic acceleration, (*SD*_ODBA_) as a measure of overall activity level. For kittiwakes, initial data exploration indicated that there was greater relative variability in the *Z*‐axis than in the ODBA, therefore, we used standard deviation in the *Z*‐axis (*SD_Z_*) to measure activity level.

Table 1 describes the accelerometer metrics calculated from accelerometers; all of these metrics have been used in prior studies classifying animal behavior from accelerometers (Chimienti et al., 2016; Pagano et al., 2017; Shamoun‐Baranes et al., 2012). Murre classifications also used depth to identify periods of diving. We calculated pitch and dynamic acceleration using a 2‐s moving window (Shepard, Wilson, Halsey, et al., 2008) and WBF using a 5‐s window, for both species. Once accelerometer statistics were calculated, we subsampled all data to 1 s intervals to reduce processing time during classification, and because our behaviors of interest occurred at intervals >1 s. All summary statistics are reported as mean ± *SD*.

### 2.3 Accelerometer track segmentation

We used a behavior‐based track segmentation approach for classification (Bom et al., 2014; Collins et al., 2015). Cliff‐nesting murres and kittiwakes must fly to travel between their nest site and foraging areas at sea, therefore, periods of flying should separate colony behavior from swimming behavior. For murres, dives are separated from flights by periods of swimming. We used this prior knowledge of seabird behavior to segment tracks into periods of consistent behavior. We first classified diving (murres) and flying (murres and kittiwakes) from the 1‐s sampled data using each method. Any behavior that occurred for <3 s was re‐assigned to the previous behavior class and each period of presumed behavior was assigned a unique segment ID. For practical reasons, we imposed a maximum length of 120 s on each segment. This ensured that if a transition between behaviors was missed, the error wold not propagate beyond 120 s. This upper limit also ensured that each type of behavior was represented proportionally in the data. Incubation bouts typically last for many hours, while bouts of flying or diving could last seconds or minutes, so although most of the birds spend a majority of their time at the nest, there would be relatively few bouts of colony behavior relative to other types of behavior. Within each segment, we recalculated movement metrics using mean pitch and mean dynamic acceleration.

### 2.4 Accelerometer classification—supervised

We used three supervised classification methods: histogram segregation (HS), random forests (RF), and NN.

#### 2.4.1 Histogram segregation

We adapted a HS approach from Collins et al. (2015). We used density plots to visualize the distribution of each variable sequentially. Characteristic peaks and valleys in the distribution were used to identify break points for different behaviors. Each behavior was classified using a stepwise approach, once the locations had been assigned to a behavior these locations were not considered for the next variable. We first classified “diving” (murres only) and “flying” using depth and WBF. Accelerometer data were then broken down into segments of continuous behavior and we calculated average pitch and average dynamic acceleration within each segment. Remaining “unknown” segments were classified to “swimming” and “colony” based on peaks in histograms for these two variables. Each track was classified individually.

#### 2.4.2 Neural network

We used the classifications from the HS method to train the NN models. We did not use the GPS data for training the model because we wanted to test classification approaches that could be applied when GPS data are not available for model training. We randomly chose ten tracks for each species, then, randomly selected 1,000 data points within each behavior class from each of these tracks to make a training dataset. This trained model was used to predict classifications for all tracks within each data set. NN models were run with five hidden nodes using the R Package “nnet,” version 7.3–12 (Venables & Ripley, 2002).

#### 2.4.3 Random forest (RF)

The random forest (RF) method used the same training data set described above for the NN model. We ran the RF models using the R package “randomForest,” version 4.5‐14 (Liaw & Wiener, 2002).

### 2.5 Accelerometer classification—unsupervised

We also used three unsupervised classification methods: KM cluster analysis, EM, and HMM. For each method, we ran analysis with between three and six classes and visually examined the classifications to decide on the number of classes that best identified the behaviors of interest. When we identified more than three (kittiwakes) or four (murres) behavior classes, classes were grouped into the behaviors of interest based on expected patterns in behavior.

#### 2.5.1 k‐Means

The KM classification was performed in two steps. For murres, dives were identified manually by classifying all data with depth below −1 m as “diving.” A KM classification was performed on WBF to identify two classes, and the class with higher WBF was labelled as “flying.” We then segmented all data into bouts of “diving” (murres only), “flying” and “unknown” behavior. Within segments of continuous behavior, we calculated the average pitch and dynamic acceleration. A second KM classification was performed on the remaining “unknown” segments with average pitch and dynamic acceleration as input variables. We used the natural logarithm of dynamic acceleration, and both variables were scaled to their range prior to analysis. The KM classification was performed on all tracks at once. Analysis was run using the “kmeans” function in base R.

#### 2.5.2 Expectation maximization

The EM classification was performed in two steps. For murres, dives were identified manually by classifying all data with depths below −1 m as “diving.” An EM classification was performed on WBF to identify two classes; the class with higher WBF was labelled as “flying.” We then segmented all data into bouts of “diving” (murres only), “flying” and “unknown” behavior. Within segments of continuous behavior, we calculated the average pitch and dynamic acceleration. A second EM classification was performed on the remaining “unknown” segments, with average pitch and dynamic acceleration as input variables. We used the natural logarithm of dynamic acceleration, and both variables were scaled to their range prior to analysis. EM classification was performed on all tracks for each species at once EM analysis was conducted using the R package “Rmixmod” package, version 2.1.1 (Langrognet, Lebret, Poli, & Iovleff, 2016). We considered Gaussian models with free proportions; BIC was used to identify the best model.

#### 2.5.3 Hidden Markov models

Hidden Markov models require data that are sampled at equal intervals, for this reason, we did not use the track segmentation approach described above. Instead, average accelerometer values for WBF, pitch, dynamic acceleration and depth were taken for 5‐s intervals (murres) and 10‐s intervals (kittiwakes). A shorter interval was used for murres to preserve short inter‐dive bouts. We used the R package “momentuHMM” (McClintock & Michelot, 2018) to fit HMMs. For murres, depth data were converted to a binary variable, where data with depth <−1 m received a value of 1 and depths >−1 received a value of 0, this was modelled using a Bernoulli distribution. A full description of the distributions and starting values used for each behavior and variable is provided in Tables 2 and 3. We fixed transition probabilities between colony‐swimming, swimming‐colony, colony‐diving, diving‐colony, diving‐flying, and flying‐diving to zero. The most likely behavioral states were obtained from the model using the Viterbi algotrithm (McClintock & Michelot, 2018).

**Table 2:** Starting values for the state‐dependent probability distribution parameters for variables used in the hidden Markov model to classify behavior of thick‐billed murres

Variable	Family	Link	Parameter	Colony	Diving	Flying	Swimming
Pitch	Normal	Identity	Mean	30	−5	0	−5
		Log	*SD*	20	50	5	10
*SD*_ODBA_	Exponential	Log	Rate	25	5	2.5	5
WBF	Log normal	Identity	Location	0.5	2	9	2
		Log	Scale	0.5	0.5	0.2	0.5
		Logit	Zero‐mass	0.9	0.9	0.1	0.9
Depth	Bernoulli	Logit	Probability	1 × 10^−12^	1 − (1 × 10^−12^)	1 × 10^−12^	1 × 10^−12^

**Table 3:** Starting values for the state‐dependent probability distribution parameters for variables used in the hidden Markov model to classify behavior of black‐legged kittiwakes

Variable	Family	Link	Parameter	Colony 1	Colony 2	Flying	Swimming
Pitch	Normal	Identity	Mean	35	10	0	5
		Log	*SD*	10	10	5	5
*SD_Z_*	Log normal	Identity	Location	0.05	0.05	0.6	0.15
		Log	Scale	0.5	0.5	0.5	0.5
		Logit	Zero‐mass	0.9	0.9	0.1	0.1
WBF	Log normal	Identity	Location	0.5	2	9	2
		Log	Scale	0.5	0.5	0.2	0.5
		Logit	Zero‐mass	0.9	0.9	0.1	0.9

### 2.6 GPS classification

#### 2.6.1 Thick‐billed Murre

We used GPS and depth sensor data to validate murre behavior classifications. Locations requiring a calculated ground speed >30 m/s were excluded from analysis (0.1% of all GPS locations), because these were potential GPS errors. If depth was below −1 m, data were labelled as diving. Remaining locations with a calculated ground speed >2 m/s were classified as flying. At relatively high sampling rates (i.e., <100 s), like those used in this study, the calculated ground speed and instantaneous speeds are expected to be highly correlated (Elliott, Chivers, et al., 2014). Locations within 250 m of the nest were classified as colony and all remaining locations were classified as swimming. Following this initial classification, each bout of continuous behavior was assigned a unique identifier. Data were examined for obvious classification errors based on temperature, duration of behavior, and behavioral context (prior and subsequent behaviors). Swimming bouts within 3 km of the colony with a high average temperature (>10°C) were examined as potential colony locations and colony bouts with low average temperature were examined as potential swimming locations. Only 0.6% of GPS locations were manually reclassified.

Because the GPS data were collected at a lower temporal resolution (60 s for murres and 30 s for kittiwakes) than the accelerometer analysis (1 s), the GPS classification would be slower to respond to a change in behavior. For example, a murre that transitions from flying to swimming halfway between two GPS fixes would be classified as still flying during the next location, however the accelerometer could pick up this change in behavior at the time it occurred. To deal with this difference in sampling rate, we identified periods when the GPS indicated a transition from one behavior to another. All data points within 60 s of a GPS transition between colony, flying, or swimming were labelled as transitions and excluded from further analysis. Transitions between diving and swimming were not excluded, because the pressure sensor collected depth data at 1 s intervals. In total, 11.0% of GPS locations were excluded for murres because they were identified as periods of transition between behaviors.

#### 2.6.2 Black‐legged Kittiwake

GPS data were used to validate kittiwake behavior classifications. Locations requiring a ground speed >20 m/s or more than 10‐min between fixes were excluded from analysis (0.4% of locations), because these were potential GPS errors. Locations with a calculated ground speed >3 m/s were classified as flying. Locations on Middleton Island were classified as colony, and all remaining locations were classified as swimming. Kittiwakes can spend significant time on tidal flats and sand bars around Middleton Island (K. Elliott, personal observation); in these locations, birds could be swimming or loafing depending on tide heights and these behaviors could not be differentiated based on the GPS data alone. Therefore, we excluded all locations within 500 m of the island from the analysis. This reduced the total GPS data set by 11.1%; this step was important to minimize uncertainty and potential for errors in our validation data. Similar to murres, all locations within 30 s of a change in behavior were labelled as transitions (13.5%) and excluded from the analysis.

### 2.7 Classification accuracy

We subsampled the accelerometer data to 1 min (murres) and 30 s (kittiwakes) to match the resolution of the GPS data and used a confusion matrix to calculate the overall accuracy and the balanced accuracy for each behavior. Confusion matrices and measures of accuracy were calculated using the R package *carat* (Kuhn, 2016). We used mixed‐effects models, with bird identity as a random effect, to test for differences in the classification accuracy among methods and between breeding stages. Accuracy data were logit transformed prior to analysis. We used the R package *nlme* (Pinheiro, Bates, DebRoy, Sarkar, & R Core Team, 2018) to run the models and the *lsmeans* package (Lenth, 2016) to calculate parameter estimates, 95% confidence intervals (CI) and for pairwise comparisons.

### 2.8 Daily energy budget

We used an estimate of DEE to look at the overall variation among classification methods. DEE (in kJ/d) for murres was calculated following Elliott et al. (2013) as:

$$\text{DEE}=32.0*t_{\text{c}}+532.8*t_{\text{f}}+100.8*t_{\text{s}}+97.2*t_{\text{d}}.$$

Daily energy expenditure for kittiwakes was calculated following Jodice et al (2003), using activity specific metabolic rates for nest attendance, commuting flight, and surface feeding to develop the equation:

$$\text{DEE}=21.0*t_{\text{c}}+99.9*t_{\text{f}}+25.8*t_{\text{s}},$$

where *t* is time in hours and the subscripts are c = colony, f = flying, s = swimming, and d = diving. We converted metabolic rates from CO_2_ production rates (ml CO^2^ g^−1−^hr^−1^) to kJ using an energetic equivalent of 27.33 kJ L CO_2_ assuming average kittiwake mass of 416 g (Jodice et al., 2003; Speakman, 1997). We used mixed effects models, with bird ID as random effects, to test for differences in DEE estimates among methods.

### 2.9 Variable selection

We chose 42 accelerometer statistics used in previous studies (Appendix S1) to consider in our variable selection analysis; these included raw acceleration values, static acceleration, dynamic acceleration, minimum, maximum, range, skew, and kurtosis for each axis. We also calculated the trend, as the slope coefficient from a linear regression, and autocorrelation, as the value of the first order autocorrelation function. Each of these statistics was calculated over a 2‐s moving window. Finally, we included the dominant frequency for each axis calculated over a 5‐s moving window.

We used random forests models to identify which variables contributed the most to classification accuracy and how much adding additional variables improved accuracy. To simulate a realistic training data set, acquired through paired GPS‐accelerometer deployments, we trained and tested data from the classified GPS tracks using a random subset of 10 individual tracks for each species. From these tracks, we sub‐sampled 1,000 locations from each behavior class to ensure each behavior was adequately represented in the training data. We used forward selection to identify which accelerometer variables provided the greatest improvement in classification accuracy for models with between 1 and 10 variables. To reduce overall computation time, variable importance from a global model with all variables and all training data were used to identify the 20 most influential variables to include in the variable selection analysis. At each step, we ran 100 simulations with randomly selected training data sets and selected the variable with highest median accuracy over all simulations. We compared model accuracy among the best models with 1–10 variables and a global model with all 42 variables. Confidence intervals for model accuracy are based on the 2.5th and 97.5th percentile of all simulations.

## 3 RESULTS

### 3.1 Classification summary

#### 3.1.1 Murres

**Figure 1:**
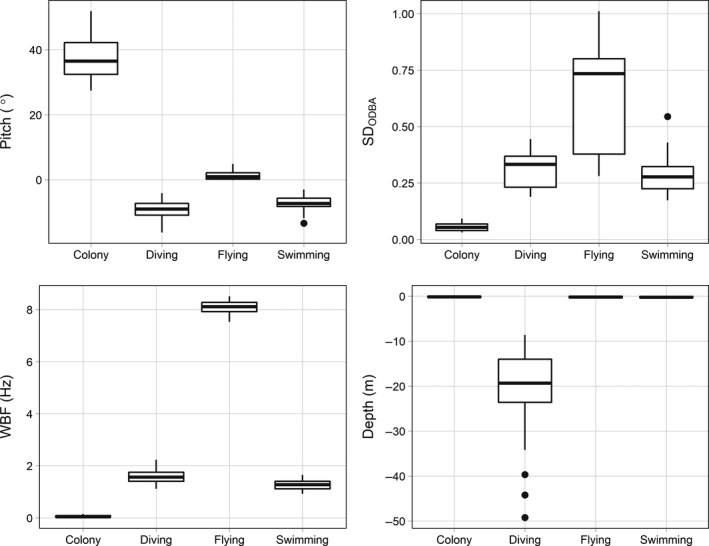
Boxplots showing the distribution of average values of predictor variables for each thick‐billed murre behavior

**Figure 2:**
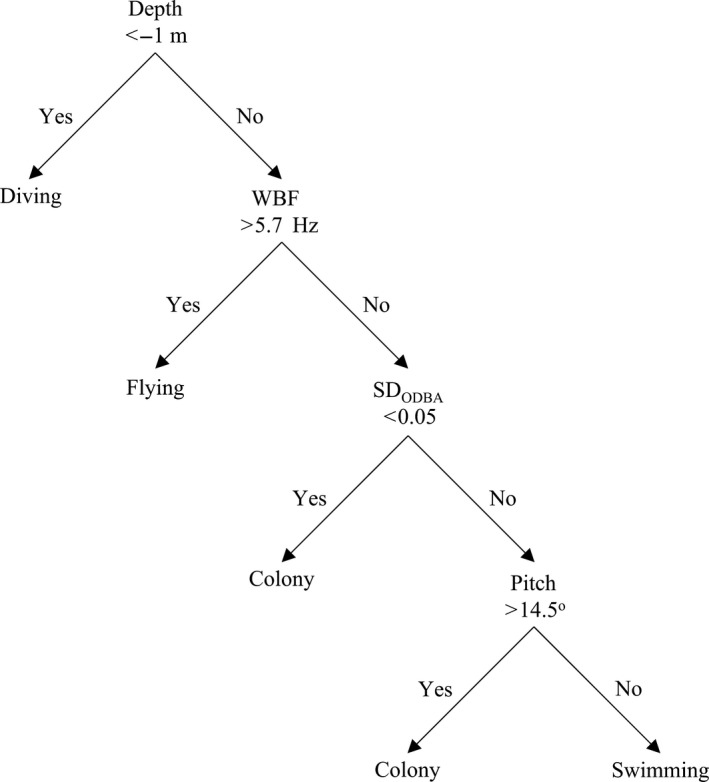
Diagram showing the average break points and classification hierarchy used in the histogram segregation method for thick‐billed murres

Colony segments were characterized by high pitch (37.6 ± 6.1°; Figure 1) and low *SD*_ODBA_ (0.05 ± 0.02 g). Swimming segments were characterized by low pitch (−7.4 ± 2.5°) and high *SD*_ODBA_ (0.28 ± 0.08 g). Flying segments had high WBF (8.1 ± 0.25 Hz). Diving segments were characterized by depths below −1 m (−20.5 ± 9.0 m). Figure 2 shows the hierarchical process and average breakpoints used for assigning behaviors with the HS method. We used five total classes in the KM classification for murres: two colony, one diving, one flying, and one swimming class. For the EM and HMM classes only four classes were necessary to obtain a clear separation of all four behaviors, based on visual examination of the classifications.

#### 3.1.2 Kittiwakes

**Figure 3:**
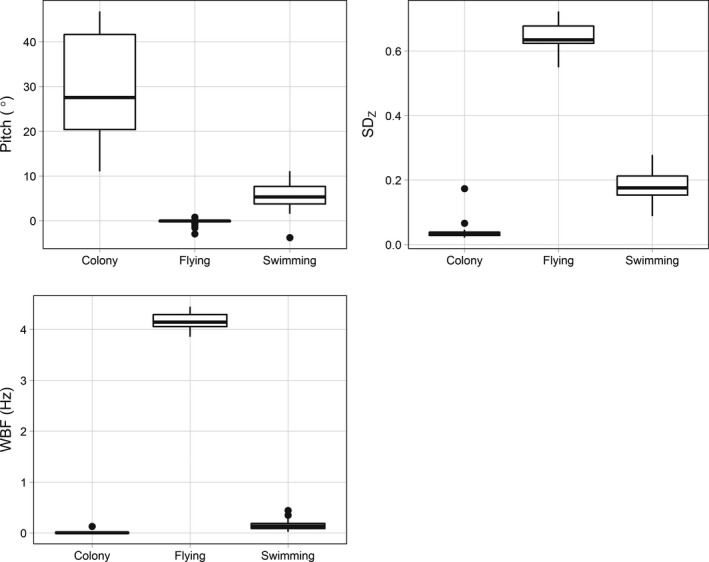
Boxplots showing the distribution of average values of predictor variables for each black‐legged kittiwakes behavior

**Figure 4:**
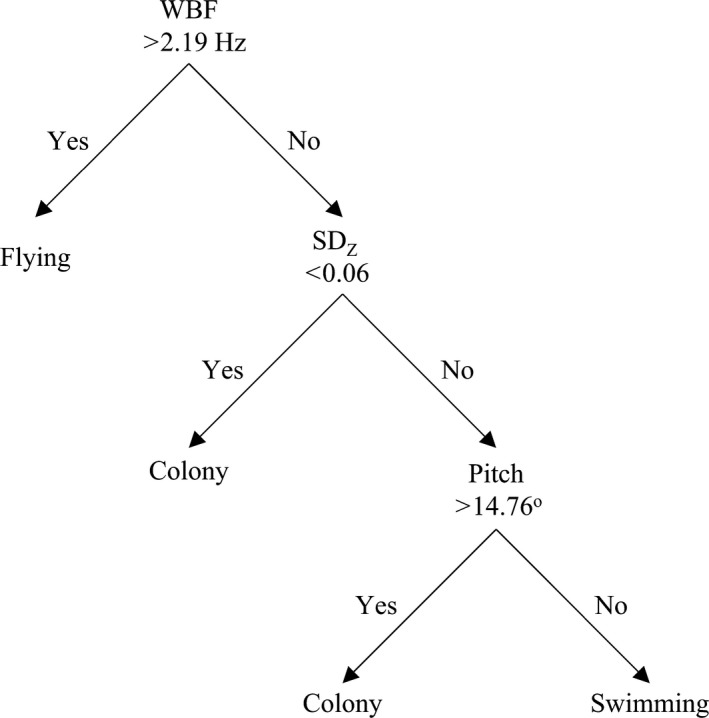
Diagram showing the average break points and classification hierarchy used in the histogram segregation method for black‐legged kittiwakes

Colony segments were characterized by high pitch (29.9 ± 11.7°; Figure 3) and low *SD_Z_* (0.04 ± 0.02 g). Swimming was characterized by low pitch (5.7 ± 2.9°) and high *SD_Z_* (0.18 ± 0.04 g). Flying segments had high WBF (4.16 ± 0.16 Hz). The HS method began by classifying flight with WBF, then colony with *SD_Z_*, and finally swimming with pitch (Figure 4). We used four total classes in the KM, EM, and HMM classifications for kittiwakes: two colony classes, one flying class, and one swimming class.

### 3.2 Classification accuracy

#### 3.2.1 Murres

**Figure 5:**
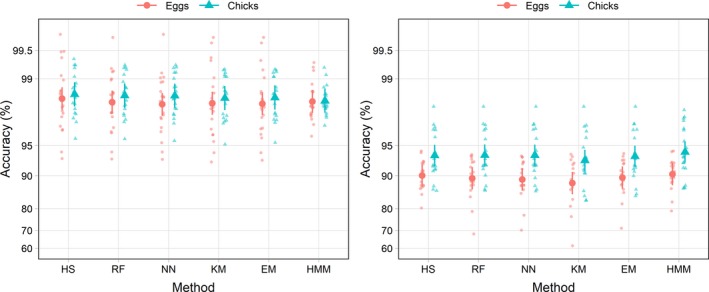
Average accuracy of classification methods for thick‐billed murre (left) and black‐legged kittwakes (right). Large symbols show group means and error bars are 95% confidence intervals, small symbols are data from each individual. Data are displayed on a logit scale

Mean classification accuracy for each method was >98.3% and accuracy for each individual track was above 92.7% for all methods (Figure 5). There was no statistical support for a difference in accuracy among classification methods (*F*_5,190_ = 1.28, *p* = 0.28). Averaging across breeding status, accuracy was highest using the HS (98.5%; CI = 98.1–98.7) method and lowest for the HMM (98.3%; CI = 97.9–98.6) method, but this difference was not statistically significant (*t*_190_ = 2.162, *p* = 0.26). Accuracy for all methods was higher for murres with chicks (98.4%, CI = 97.9–98.8) than for murres with eggs (98.2%, CI = 97.7–98.6); however, there was no evidence that accuracy varied with breeding status (*F*_1,38_ = 0.46, *p* = 0.50) or for an interaction between method and breeding status (*F*_5,190_ = 0.75, *p* = 0.58).

**Figure 6:**
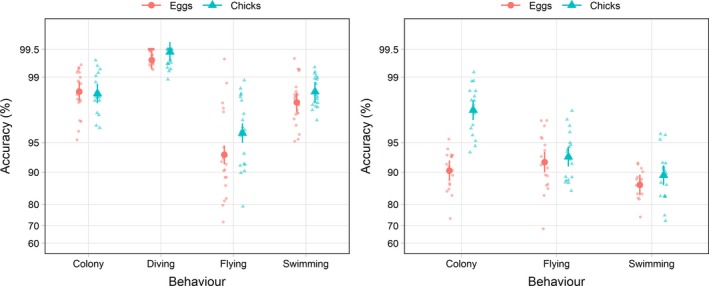
Average accuracy for thick‐billed murre (left) and black‐legged kittwakes (right) behaviors; only results from the histogram segregation (HS) method are shown. Large symbols show group means and error bars are 95% confidence intervals, small symbols are data from each individual. Data are displayed on a logit scale

There was a significant interaction between method and behavior (*F*_15,894_ = 23.6, *p* < 0.001; Figure 6), indicating that some methods were more accurate at classifying certain behaviors than other methods. Average classification accuracy for colony across all methods was 99.1% (CI = 99.5–99.7); there were no significant differences in classification accuracy among methods for colony (all *p* > 0.35). Average classification accuracy for swimming across all methods was 98.7% (CI = 98.4–98.9); there were no significant differences in classification accuracy among methods for swimming (all *p* > 0.06). The HMM method was most accurate for classifying flying (97.9%, CI = 97.4–98.3); this was significantly higher than all other methods (NN: 95.3%, CI = 94.3–96.2; *t*_894_ = 6.88, *p* < 0.001; HS: 95.3%, CI = 94.3–96.2; *t*_894_ = 6.93, *p* < 0.001; RF: 95.3%, CI = 94.3–96.2; *t*_894_ = 6.97, *p* < 0.001; EM :94.4%, CI = 93.1–95.4; *t*_894_ = −8.58, *p* < 0.001; KM: 94.3%, CI = 93.1–95.4; *t*_894_ = 8.69, *p* < 0.001). For diving, classification accuracy was highest for the HS (99.9%; CI = 99.8–1.00), EM (99.9; CI = 99.8–1.00), and KM (99.9%; CI = 99.8–1.00) methods, and lowest for the HMM method (98.2%; CI = 97.8–98.6). High classification accuracy for diving is expected, because dives in the GPS data and accelerometer data were both classified using the depth sensor. There was a significant interaction between behavior and stage (*F*_3,894_ = 15.9, *p* < 0.001). Flying was classified more accurately during chick rearing (96.7%, CI = 95.9–97.4) than during incubation (94.2%, CI = 92.9–95.2; *t*_38_ = 3.92, *p* < 0.001) and there was some evidence that swimming was classified more accurately during chick rearing than incubation (*t*_38_ = −1.91, *p* = 0.06).

#### 3.2.2 Kittiwakes

There was strong evidence for a difference in classification accuracy among methods (*F*_5,170_ = 6.21; *p* < 0.001) and between breeding stages (*F*_1,34_ = 9.41; *p* = 0.004), there was no support for an interaction between method and breeding stage (*F*_5,170_ = 0.41; *p* = 0.84; Figure 5). Averaging across all methods, accuracy during the chick stage was 93.7% (CI = 92.0–95.0) while accuracy was 89.5% (CI = 86.7–91.7) during the egg stage. For birds with eggs, there were no significant differences among the HMM (90.3%, CI = 87.7–92.5), HS (90.0%, CI = 87.3–92.3), EM (89.6%, CI = 86.7–91.9), RF (89.4%, CI = 86.5–91.8), and NN (89.1%, CI = 86.2–91.5) methods. The KM (88.2%, CI = 85.1–90.8) method was significantly less accurate than the HMM (*t*_170_ = −3.87, *p* = 0.002) and HS (*t*_170_ = −3.25, *p* = 0.02) methods. The absolute difference in accuracy between the most accurate method, HMM, and the least accurate method, KM, was only 2.1%. During the chick rearing stage, there were no differences in classification accuracy among the HMM (94.2%, CI = 92.6–95.4), RF (93.7%, CI = 92.0–95.1), NN (93.7%, CI = 92.0–95.1), HS (93.7%, CI = 92.0–95.1), and EM (93.6%, CI = 91.8–95.0) methods. Classification accuracy for the KM method (93.0%, CI = 91.0–94.5) was significantly lower than the HMM (*t*_170_ = −3.75, *p* = 0.003) method. The absolute difference in accuracy between the best and worst classification methods was only 1.2%.

There was no interaction between method and behavior (*F*_10,593_ = 0.66; *p* = 0.77), indicating that all methods classified different behaviors with similar accuracy. There was a significant interaction between behavior‐specific accuracy and breeding stage (*F*_2,593_ = 163.0; *p* < 0.001; Figure 6). Colony behavior was identified more accurately during the chick stage (97.6%, CI = 97.1–98.1) than during the egg stage (90.0%, CI = 87.8–91.8; *t*_34_ = −10.3; *p* < 0.001). There was no difference in classification accuracy for swimming across stages (Eggs: 92.2%, CI = 90.5–93.7; Chicks: 93.1%, CI = 91.7–94.4; *t*_34_ = −1.632, *p* = 0.11). There was also no difference in accuracy of flight classification between stages (Eggs: 88.5%, CI = 83.0–88.3; Chicks: 88.5%, CI = 86.3–90.5; *t*_34_ = −0.92, *p* = 0.37).

### 3.3 Daily energy budget

#### 3.3.1 Thick‐billed murres

There was a significant difference in estimates of DEE among methods (*F*_5,190_ = 40.3, *p* < 0.001) and suggestive evidence of an interaction between method and breeding status (*F*_5,190_ = 2.19, *p* = 0.06). For murres with eggs, mean DEE calculated with the RF classification (2,112 kJ/day, CI = 1,908–2,315) was lower than DEE calculated with all other methods (EM 2,242 kJ/day, CI = 2,038–2,446, *t*_190_ = −8.76, *p* < 0.001; HS: 2,242 kJ/day, CI = 2,038–2,446, *t*_190_ = −8.76, *p* < 0.001; KM: 2,242 kJ/day, CI = 2,038–2,446, *t*_190_ = −8.77, *p* < 0.001; HMM: 2,265 kJ/day, CI = 2,061–2,468, *t*_190_ = −10.3, *p* < 0.001; NN: 2,272 kJ/day, CI = 2,069–2,476, *t*_190_ = −10.8, *p* < 0.001). During incubation, the difference between average DEE estimate using the RF method and the NN method, was only 161 kJ or 7.1% of mean DEE. During chick rearing, mean DEE calculated using the RF (2,375 kJ/day, CI = 2,161–2,589) classification was significantly lower than all other methods (KM 2,454 kJ/day, CI = 2,240–2,669, *t*_190_ = −5.06, *p* < 0.001; EM: 2,455 kJ/day, CI = 2,240–2,669, *t*_190_ = −5.06, *p* < 0.001; HS: 2,455 kJ/day, CI = 2,241–2,669, *t*_190_ = −5.11, *p* < 0.001; HMM: 2,471 kJ/day, CI = 2,257–2,685, *t*_190_ = −6.11, *p* < 0.001; NN: 2,475 kJ/day, CI = 2,260–2,689, *t*_190_ = −6.36, *p* < 0.001). The difference between average DEE estimate during chick rearing using the RF method and the NN method, was only 99 kJ or 4.0% of mean DEE.

#### 3.3.2 Kittiwakes

Breeding status had a significant effect on DEE (*F*_1,37_ = 23.5, *p* < 0.001); kittiwakes with chicks (1,222 kJ/day, CI = 1,116–1,327) had significantly higher DEE than kittiwakes with eggs (869 kJ/day, CI = 767–972). Classification method had a significant effect on estimates of DEE (*F*_5,185_ = 74.8, *p* < 0.001). During incubation, the RF method had significantly lower estimates of DEE (842 kJ/day, CI = 739–944) than all other methods (NN: 874 kJ/day, CI = 771–977, *t*_185_ = −9.09, *p* < 0.001; KM: 875 kJ/day, CI = 772–978, *t*_185_ = −9.31, *p* < 0.001; HS: 875 kJ/day, CI = 772–978, *t*_185_ = −9.32, *p* < 0.001; HMM: 875 kJ/day, CI = 772–978, *t*_185_ = −9.35, *p* < 0.001; EM: 876 kJ/day, CI = 773–977, *t*_185_ = −9.63, *p* < 0.001). However, the difference between average DEE estimates during incubation using the RF method and the EM method, which had the highest average DEE estimates, was only 34 kJ or 3.9%. For kittiwakes with chicks, the RF method (1,185 kJ/day, CI = 1,080–1,291) also had significantly lower estimates of DEE than all other methods (HMM: 1,229 kJ/day, CI = 1,123–1,334, *t*_185_ = −9.09, *p* < 0.001; HS: 1,229 kJ/day, CI = 1,123–1,334, *t*_185_ = −9.31, *p* < 0.001; KM: 1,229 kJ/day, CI = 1,223–1,334, *t*_185_ = −9.32, *p* < 0.001; EM: 1,229 kJ/day, CI = 1,123–1,334, *t*_185_ = −9.35, *p* < 0.001; NN: 1,229 kJ/day, CI = 1,224–1,335, *t*_185_ = −9.63, *p* < 0.001). During chick rearing, the difference between average DEE estimate using the RF method and the NN method, was only 44 kJ or 3.6% of mean DEE.

### 3.4 Variable selection

#### 3.4.1 Thick‐billed murres

**Figure 7:**
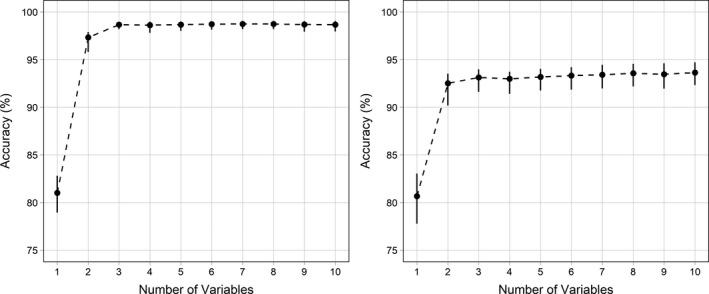
Change in thick‐billed murre (left) and black‐legged kittiwake (right) behavior classification accuracy with additional variables included in random forest models using a forward selection procedure. Black points are medians and error bars are 95% confidence intervals

Classification accuracy increased from the best possible model using a single variable, 81.0% (CI = 78.7–82.3) to 98.7% (CI = 98.2–98.9) accuracy for the best model using three variables. Adding more than three variables to the model did not increase model accuracy (Figure 7). Variable selection identified WBF, depth, and static*_X_*, as the three variables with the greatest influence on classification accuracy. A global model using all 43 candidate variables had 98.8% (CI = 98.2–99.1) classification accuracy, which overlaps the accuracy achieved with the three‐variable model. Following the same procedure using our original variables, WBF, pitch, depth, and sdODBA, gave comparable accuracy at 98.5% (97.7–98.9). Pitch, one of our a priori variables, was the fifth variable after static*_X_* and skew*_Z_*. Static*_X_* and pitch had a correlation coefficient of 0.96 (CI = 0.964–0.965; *p* < 0.001); therefore, these two variables may be largely interchangeable. Our chosen measure of dynamic acceleration, *SD*_ODBA_, did not rank among the twenty most important variables, indicating that including this metric in our original models may not have contributed to classification accuracy.

#### 3.4.2 Kittiwakes

Classification accuracy increased from the best possible random forest model using a single variable, 80.7% (CI = 77.8–83.0), to the best model using two variables, 92.5% (CI = 90.2–93.5). Additional variables did not substantially increase model accuracy (Figure 7). A global model using all 42 candidate variables had 93.4% (CI = 91.7–94.6) classification accuracy, which overlaps the accuracy achieved with the best two‐variable model. Forward selection identified auto‐correlation in the *Z*‐axis (ACF*_z_*) and WBF as the best predictors in a two‐variable model. ACF*_Z_* had low values during colony segments (0.1 ± 0.1), intermediate values during flying segments (0.5 ± 0.04), and high values during swimming segments (0.7 ± 0.12). As with the initial classification methods, WBF was high during periods of flight and low during periods of swimming or periods on the colony.

Our original model using WBF, pitch and *SD_Z_* had comparable accuracy, 92.5% (CI = 90.4%–93.6%), to the top two variable model identified through variable selection. ACF*_Z_* appeared to measure differences in activity in kittiwake behavior that were not apparent in pitch or *SD_Z_*. For both pitch and *SD_Z_*, average values of pitch and *SD_Z_* for colony were more similar to swimming than flying, while average values of ACF*_Z_* for colony and swimming were more distinct than from average values for flying. Since our original model had lower accuracy for swimming and colony behavior, at least during incubation, ACF*_Z_* may provide better classification for these behaviors.

## 4 DISCUSSION

We found high classification accuracy using a small number of accelerometer‐derived metrics to identify coarse‐scale animal behavior. Accuracy was robust to choice of classification method. Although there were statistically significant differences in classification accuracy for the methods tested, average accuracy of all methods was high (98% murres, 91% kittiwakes). There were no differences in mean accuracy among methods for murres and relatively small differences in mean accuracy among methods for kittiwakes. Choice of classification method appears to have little impact on classification results. Any of the methods described here should provide a robust classification of the principal behavior types for murres and kittiwakes. We expect these results to be largely transferable to other species in the same families, and potentially more broadly applicable to other waterbirds that use flapping flight.

We were able to achieve highly accurate and consistent results across all methods using a small set of predictor variables. For both species, including more than two or three predictor variables gave no significant improvement in classification accuracy. Many other studies, particularly those using machine learning methods, include large numbers of predictor variables (Ladds et al., 2017; Nathan et al., 2012). We found that limiting the number of variables greatly reduced analysis time, because files are smaller and models are simpler. Resulting classifications are easier to interpret, especially for unsupervised classifications, because they are based on fewer predictors with an a priori relationship to behavior.

More importantly, we have shown that similar variables–pitch, dynamic acceleration, and WBF–can be used to classify the behavior of two different seabird species. The predictor variables we selected are likely to be useful in classifying coarse‐scale behaviors for a wide range of species, because changes in pitch, dynamic acceleration, and periodicity are fundamental components of all activity (Shepard, Wilson, Quintana, et al., 2008). Even in non‐flying species, locomotion (walking, running, and swimming) should have a distinct signature in the frequency domain which would help identify this type of behavior (Shepard, Wilson, Quintana, et al., 2008). Measures of pitch, dynamic acceleration, and frequency should be a good starting point in any behavioral classification. However, our variable selection identified another variable, ACF*_Z_* for kittiwakes, which performed slightly better in classifying behavior for this species, the difference in average accuracy in using this variable was minimal. In the absence of training data to conduct similar variable selection, the types of accelerometer statistics we selected a priori for our models are likely to be effective in classifying basic behavior for a range of species.

That classification accuracy was consistently high is perhaps not a surprising result. Many studies have found higher accuracy when only a small number of general behaviors is considered (Hammond, Springthorpe, Walsh, & Berg‐Kirkpatrick, 2016; Ladds et al., 2017; Shamoun‐Baranes et al., 2012). Indeed, the behaviors we considered are readily identifiable in an accelerometer trace using the human eye. The challenge for researchers is developing methods that can automatically, and reliably, label these behaviors. This study is notable because we have demonstrated that these behaviors are easily identifiable using large data set from two different, wide‐ranging seabird species, which cannot be easily observed in the wild.

Our classification of murre behavior benefitted from incorporating data from a pressure sensor to measure depth and identify dives. However, the behavior specific accuracy for the other three behaviors (colony, flying, and diving) were all >94%, so even if diving was excluded the overall classification accuracy for murres would have been high using our methods. Pressure sensors add little to the weight and size of an accelerometer, so for most diving species there is no reason not record pressure data along with acceleration. For very small diving species, further development of methods to classify dives and estimate depth using only accelerometer data are needed.

Classification accuracy is not the only factor that should influence choice of classification method. Depending on the research questions being addressed, certain methods may be more appropriate. Hidden Markov models offer advantages, beyond high classification accuracy, that are not achieved with the other methods considered here. Specifically, HMMs account for the serial dependence in an acceleration time series (Leos‐Barajas et al., 2016). In this study, we could directly model the expected transitions between our three or four behavioral states by setting priors on the transition probabilities. Indeed, for the other classification methods we used a track segmentation approach to improve our ability to detect broad scale behaviors. Our segmentation approach would not work for species that do not have to transition through one behavior (e.g., flight) to begin another behavior. HMMs can also be used to jointly model how external factors influence behavior (Leos‐Barajas et al., 2016). Using other methods, this must be done in as a two‐step process, first classifying behavior and then testing for relationships with external factors. However, the HMMs are arguably the least accessible method we considered; they require sophisticated statistical understanding to implement, and success in behavioral classification depends on carefully specified priors. For applications where behavioral classification is likely to be high, and data will ultimately be summarized at large timescales (e.g., hours, days, or longer), the advantages of using HMMs may not outweigh the costs of implementing this method.

Our methods worked across two different species and breeding stages (incubation vs. chick‐rearing). Nonetheless, classifications were more accurate with murres than kittiwakes across all methods. Murres have high wing loading and high wing beat frequencies (Elliott et al., 2013; Pennycuick, 1987). As a result, murres only use flapping flight, which is easily defined from accelerometer profiles. Kittiwakes have much lower wing loading and lower wing beat frequencies (Jodice et al., 2006; Pennycuick, 1987). Murres make rapid, directed flights with few landings on the water, which helps to distinguish flight from swimming in GPS tracks. The more agile kittiwakes change direction and make short, frequent landings while visually searching for prey, which would create more overlap in ground speeds measured by GPS. Simultaneous deployments of GPS‐accelerometers with salinity loggers or a magnetometer could help improve validation of kittiwake behavior classifications and identify accelerometer measures characteristic of gliding flight.

In principle, there should be no difference in the behaviors we classified between incubation and chick rearing, because all of these behaviors occur in all stages of the annual cycle. However, we did find it was more difficult to classify swimming and colony behavior accurately for incubating kittiwakes than for chick‐rearing kittiwakes. For both species, swimming was primarily differentiated from colony using differences in dynamic acceleration and pitch. Kittiwakes build a nest structure to hold their eggs and can be quite active in shifting positions and turning eggs within their nest cup. This activity at the nest and changes in pitch during incubation may have made it more difficult to differentiate incubation from swimming consistently. Additionally, during incubation kittiwakes may spend more time resting on the water, which would have relatively low dynamic acceleration compared to active foraging on the water, making it more difficult to discern from time spent at the nest. Variable selection analysis found that ACFz was a stronger predictor of behavior for kittiwakes than either pitch or *SD_Z_*. ACF*_Z_* showed strong differentiation between swimming and colony, making it potentially a more useful variable in classifications for kittiwakes.

For any behavioral classification, the position of the data logger on the animal could influence the utility of certain acceleration measures. For example, a logger mounted on the tail or legs would have a different pitch signature than a logger mounted on the back or stomach, and may show different patterns of dynamic acceleration from the main body. Additionally, variation in how loggers are attached to individual animals can influence the ability to identify different behaviors between tracks. Indeed, in our data the differences in classification accuracy among individuals was significantly larger than the differences in classification accuracy among methods. Therefore, there should be careful consideration of logger position, and consistency in logger attachment, during study design, implementation, and data analysis.

By using a training data set for the RF and NN methods that only included a sub‐sample of individuals, we demonstrated that data from a small number of individuals was transferable to a larger sample of individuals. Acquiring training data for wide‐ranging species like seabirds is an impediment to using supervised classification methods for labelling behaviors. We have demonstrated that a simple supervised classification method can be used to build a training data set for basic behaviors in seabirds. The NN and RF approaches have the advantage that classifications can be fully automated without any user input once a training data set has been developed. The use of machine learning techniques for classification of wide ranging species can be limited by the challenges of developing a training data set. With large data sets, a training data set could be developed based on a subsample of data using any of the other four methods described here, and a model based on this training data could be used to classify remaining data.

Wing beat frequency was an important variable in our classifications. Estimating WBF from accelerometer data requires a sampling frequency that is at least two times higher than the expected WBF (or equivalent movement pattern) of the focal species (“the Nyquist frequency”). WBF also has many ecological applications, such as estimating changes in mass after a foraging bout (Sato, Daunt, Watanuki, Takahashi, & Wanless, 2008) and measuring changes in flight costs associated with environmental conditions (Elliott et al., 2013). Flapping flight is one of the most energetically expensive behaviors for seabirds, so accurately quantifying this behavior is important for energetic estimates. We recommend accelerometer studies on seabirds use a sampling frequency that will allow estimation of WBF, which is consistent with other authors recommendations for sampling frequencies to adequately sample dynamic body acceleration (Gómez Laich, Wilson, Gleiss, Shepard, & Quintana, 2011). For behavioral classifications, we cannot perceive any strong rationale for sampling at frequencies higher than 2–3 times the expected WBF of a focal species.

Coarse‐scale behavior identification, like the approaches demonstrated here, could be a first step in a hierarchical process of identifying fine‐scale behaviors (Leos‐Barajas et al., 2017, 2016). Several studies have been successful in distinguishing general behaviors, like the behaviors identified in this paper, but have been less successful in effectively classifying finer scale behaviors associated with prey capture, prey handling and self‐maintenance (Hammond et al., 2016; Ladds et al., 2017; Shamoun‐Baranes et al., 2012). An initial partitioning into general behavior classes may simplify the process of defining detailed behavior profiles, especially where these behaviors occur as a subset within more coarse‐scale behavior. While our results show that accurate classification of basic seabird behaviors can be developed using simple methods and a small group of accelerometer statistics, identifying fine scale behavior may require independently collected training data, and a larger suite of predictor variables, to capture the unique characteristics of less common behaviors.

## 5 CONCLUSION

Obtaining reliable activity budgets from free‐ranging animals is important for addressing a wide range of questions in wildlife ecology and animal behavior. Combined with methods for tracking animal location, behavioral classification from accelerometers could be used to examine the relationship between behavior and environmental conditions over large spatial and temporal scales. We believe that uncertainty about how to classify behavior from accelerometers has been a barrier to wider use of this technique. Our results demonstrate that general behaviors of seabirds can be classified from acceleration profiles using a range of techniques and a small number of predictor variables. Choice of classification method had a negligible effect on accuracy, therefore, researchers should not be impeded by a need to develop and apply the most advanced classification method, as multiple methods can provide similar results when classifying a small number of common behaviors. However, this finding may not hold in cases where the objective is to identify more detailed types of behavior than the broad classes considered here. Where the goal of classification is to develop a daily activity budget or estimate DEE, then simple classification methods are likely adequate, at least for waterbirds that primarily use flapping flight. Where the goal is to examine how different factors effect behavior, the HMM approach may be preferable because this approach can be used to directly test the effect of predictor variables on behavior.

## CONFLICT OF INTEREST

None declared.

## Data Availability

Data used in this analysis and R scripts for behavioral classifications have been archived at https://datadryad.org/(https://doi.org/10.5061/dryad.2hf101c).
