# Supplementary material for: A comparison of techniques for classifying behavior from accelerometers for two species of seabird
Source: Ecol Evol. 2019 Feb 21;9(6):3030–45. doi: 10.1002/ece3.4740 (PMC6434605; doi:10.1002/ece3.4740)
Supplement: Supplementary file 1 [file ECE3-9-3030-s001.docx]

Appendix. Summary of recent studies that used accelerometers to classify animal behaviours.

| Study | Species | n | Accelerometer Measures | Predictor Variables | Behaviours Identified | Classification Methods | Accuracy (%) |
| --- | --- | --- | --- | --- | --- | --- | --- |
| Gómez Laich et al 2009 | Imperial cormorant (*Phalacrocorax atriceps*) | 14 | Pitch, acceleration, standard deviation, frequency | 7 | 8 | Thresholds | - |
| Moreau et al 2009 | Goat (*Capra aegagrus hircus*) | 3 | Acceleration, inclination | 4 | 3 | Thresholds | Eating: 87 - 93 Resting: 68 - 90 Walking: 20 - 92 |
| Nathan et al 2011 | Griffon vulture (*Gyps fulvus*) | 43 | Mean, standard deviation, skewness, kurtosis, maximum, minimum, ACF, trend, square root of sum-of-squares, pair-wise correlation, ODBA, inclination, azimuth | 38 | 7 | Linear discriminant analysis (LDA), support vector machines (SVM), classification and regression trees (CART), random forests (RF), artificial neural networks (ANN) | ANN: 84.8 CART: 86.0 LDA: 86.7 RF: 90.9 SVM: 87.0 |
| Shamoun-Baranes et al 2012 | Oystercatcher (*Haematopus ostralegus*) | 3 | GPS speed, pitch, roll, maximum dynamic acceleration, mean dynamic acceleration, frequency, dominant power spectrum | 17 | 3, 8 | Classification trees | 3 Behaviours: 86.8 8 Behaviours: 73.6 |
| Bidder et al 2014 | Badger (*Meles meles*),  Camel (*Camelus dromedaries*),  Cheetah (*Acinonyx jubatus*), Imperial cormorant (*Phalacrocorax atriceps*),  Dingo (*Canus lupus dingo*), Kangaroo (*Macropus rufus*), Wombat (*Lasiorhinus latifrons*),  Human (*Homo sapiens*) | 5,  1,  1,  14,  1,  1,  1,  1 | Acceleration | 3 | 5 | K-nearest neighbour | Badger: 71 Camel: 82 Cheetah: 77 Cormorant: 77 Dingo: 83 Kangaroo: 91 Wombat: 76 Human: 95 |
| Bom et al 2014 | Crab plover (*Dromas ardeola*) | 22 | Mean, standard deviation, maximum, minimum, skewness, kurtosis, dominant power spectrum, trend, dynamic acceleration, overall dynamic body acceleration | 31 | 8 | Random forest | Inactive: 95  Fly: 89  Walk: 88  Handle: 84  Search: 78 |
| Resheff et al 2014 | Griffon vulture (*Gyps fulvus*) | nr | Mean, standard deviation, skewness, kurtosis, maximum, minimum, normalized, covariance, correlation, mean difference, standard deviation difference, dynamic acceleration, overall dynamic body acceleration, wave amplitude, line crossings, 25th percentile, 50th percentile, 75th percentile | 52 | 6 | Artificial neural network (ANN), decision tree, linear-support vector machine (LSVM), linear/quadratic discriminant analysis (LDA), nearest neighbours, radial basis function for support vector machine (RBF SVM), random forest (RF) | ANN: 84.8 Decision tree: 77.7 LDA: 80.8 LSVM: 80.1 Nearest neighbour: 80.5 RF: 84.0 RBF SVM: 82.6 |
| Berlincourt et al 2015 | Short-tailed shearwater (*Puffinus tenuirostris*) | 10 | Static acceleration, dynamic acceleration, pitch, continuous wavelet transformation, speed |  | 5 | Ethographer | - |
| Collins et al 2015 | Black-legged kittiwake (*Rissa tridactyla*) | 6 | Pitch, standard deviation | 2 | 3 | Histogram segregation | Flying: 97.9 On land: 97.5 On water: 97.5 |
| Chimienti et al 2016 | Razorbills (*Alca torda*),  Common guillemots (*Uria aalge*) | 2,  5 | Acceleration, depth, vertical speed, static acceleration, dynamic acceleration, pitch, amplitude, pitch variance | 7 | 5, 7 | Expectation maximization | - |
| Hammond et al 2016 | Alpine chipmunk (*Tamais alpinus*) Lodgepole chipmunk (*Tamais speciosus*) | 20 | Mean , variance, minimum, maximum, covariance, spectral features |  | 2-5 | Hidden semi-Markov model, optimum-threshold baseline, support vector machine learning | 2 Behaviours: 89.9 3 Behaviours: 81.6 4 Behaviours: 73.5 5 Behaviours: 73.3 |
| Leos-Barajas et al 2016 | Blacktip reef shark (*Carcharhinus melanopterus*),  Verreaux’s eagle (*Aquila verreauxii*) | 1,  1 | Overall dynamic body acceleration, minimum specific acceleration | 1 | 2 | Hidden Markov model | - |
| Cianchetti-Benedetti et al 2017 | Scopoli’s shearwater (*Calonectris diomedea*) | 60 | Vectorial dynamic body acceleration, static acceleration | 2 | 3 | Thresholds | - |
| Ladds et al 2017 | Australian fur seal (*Arctocephalus pusillus doriferus*),  New Zealand fur seal (*Arctocephalus forsteri*),  Sub-antarctic fur seal (*Arctocephalus tropicalus*),  Australian sea lion (*Neophoca cinerea*) | 2,  3,  1,  6 | Mean, median, minimum, maximum, range, standard deviation, skewness, kurtosis, absolute value, inverse covariance, trend, 10^th^ percentile, 90^th^ percentile, square root of sum of squares, pairwise correlation, inclination, azimuth, dynamic body acceleration, partial dynamic body acceleration, overall dynamic body acceleration, vectorial dynamic body acceleration | 147 | 4, 6 | Random forest, gradient boosting machine, logistic regression, super machine learning | 4 Behaviours: 85.1  6 Behaviours: 73.6 |
| Pagano et al 2017 | Polar bear (*Ursus maritimus*) | 7 | Static acceleration, dynamic acceleration, overall dynamic body acceleration, dominant power spectrum, frequency, magnitude, wet/dry status | 25 | 10 | Random forest | Resting: 97.3 Walking: 97.1 Swimming: 88.7 Running: 70.9 |
